# Effects of Dietary Carbohydrate Replaced with Wild Rice (*Zizania latifolia* (Griseb) Turcz) on Insulin Resistance in Rats Fed with a High-Fat/Cholesterol Diet

**DOI:** 10.3390/nu5020552

**Published:** 2013-02-15

**Authors:** Shufen Han, Hong Zhang, Liqiang Qin, Chengkai Zhai

**Affiliations:** 1 Department of Nutrition and Food Hygiene, School of Public Health, Southeast University, 87 Dingjiaqiao Road, Nanjing 210009, China; E-Mails: sfhan@suda.edu.cn (S.H.); phzhanghong@hotmail.com (H.Z.); 2 Department of Nutrition and Food Hygiene, School of Public Health, Medical College of Soochow University, 199 Renai Road, Suzhou 215123, China; E-Mail: qinliqiang@suda.edu.cn

**Keywords:** insulin resistance, adipocytokine, white rice and processed wheat starch, wild rice, rats

## Abstract

Wild rice (WR) is a very nutritious grain that has been used to treat diabetes in Chinese medicinal practice. City diet (CD) is based on the diet consumed by Asian area residents in modern society, which is rich in saturated fats, cholesterol and carbohydrates. The present study was aimed at evaluating the effects of replacing white rice and processed wheat starch of CD with WR as the chief source of dietary carbohydrates on insulin resistance in rats fed with a high-fat/cholesterol diet. Except the rats of the low-fat (LF) diet group, the rats of the other three groups, including to high-fat/cholesterol (HFC) diet, CD and WR diet, were fed with high-fat/cholesterol diets for eight weeks. The rats fed with CD exhibited higher weight gain and lower insulin sensitivity compared to the rats consuming a HFC diet. However, WR suppressed high-fat/cholesterol diet-induced insulin resistance. WR decreased liver homogenate triglyceride and free fatty acids levels, raised serum adiponectin concentration and reduced serum lipocalin-2 and visfatin concentrations. In addition, the WR diet potently augmented the relative expressions of adiponectin receptor 2, peroxisome proliferator-activated receptors, alpha and gamma, and abated relative expressions of leptin and lipocalin-2 in the tissues of interest. These findings indicate that WR is effective in ameliorating abnormal glucose metabolism and insulin resistance in rats, even when the diet consumed is high in fat and cholesterol.

## 1. Introduction

The intake of food rich in saturated fats, cholesterol and refined carbohydrate, which is the diet composition of Asian area residents, is pervasive and, consequently, results in the prevalence of nutrition-related chronic metabolic diseases in China, Japan and other developing countries [1,2]. Insulin resistance has been shown to be the major contributing factor to these metabolic diseases [3,4]. In addition, the dietary carbohydrate source of this diet, based on the composition of Asian area residents, is mainly from white rice and processed wheat starch, and many of the health-promoting components of intact whole-grain cereals were lost upon refining. Evidence is accumulating that consumption of high whole-grain cereals may reduce the risk of obesity, diabetes and cardiovascular diseases [5,6,7]. To date, the main approach used for exploring the physiological functions and recognized health benefits of whole-grain cereals has been focused on investigating each isolated compound. However, it is agreed to advance that this is the synergistic action of many bioactive compounds occurring in intact whole-grain cereals [8,9].

Wild rice (WR), which is grown as an aquatic grain, belongs to the genus *Zizania* with four known species. Three of these species, including *Zizania aquatica* L., *Zizania palustris* L. and *Zizania texana* Hitche, are native to North America, and only one [*Zizania latifolia* (Griseb) Turcz] is native to China, Japan and Vietnam. According to the international American Association of Cereal Chemists’ (AACC) definition, the US Food and Drug Administration published a Draft Guidance on Whole-grain Label Statements in 2006 [10]. WR belongs to whole-grain cereals. In recent years, there has been increasing consumer awareness of WR, but it has not attained regular use as a staple food. More than 3000 years ago, the grain was used in Chinese herbal medicine to treat a variety of ailments associated with nutrition [11], and Japan also had a similar record. The distribution of WR was extensive in China, but its use as a grain has completely disappeared. North American WR, which is commercialized and considered as a health food, has been widely available in supermarkets and restaurants today [12]. Composition analysis of wild rice reveals that it is rich in plant protein, dietary fiber, the amino acid lysine and low in fat [13,14,15]. Little is known about the underlying biological function of WR in improving chronic metabolic disorders. Recent animals studies have demonstrated that consuming WR can drastically improve blood lipid profiles and suppress oxidative stress [16]. 

The nutritional value and biological function of Chinese wild rice has been extensively studied in our laboratory. However, its mechanisms of action in improving glucose metabolism and insulin resistance are currently not known. Thus, we tested the hypothesis that WR as a whole-grain cereal, which replaces white rice and processed wheat starch as the chief source of dietary carbohydrates, had a beneficial effect on glucose metabolism and insulin resistance in rats fed with a high-fat/cholesterol diet. The present study also ascertained the relative expressions of peroxisome proliferator-activated receptors, alpha and gamma (PPAR-α and PPAR-γ), involved in fat storage and insulin sensitivity [17] and a series of adipocytokines, including adiponectin (ADP), leptin, lipocalin-2 (LCN2) and visfatin levels, related to insulin release and insulin resistance [18]. 

## 2. Experimental Section

### 2.1. Animals and Diets

Ten-week old male Sprague-Dawley rats were purchased from Shanghai Laboratory Animal Center (Shanghai, China). All rats were individually housed in stainless-steel cages under controlled conditions of temperature at 21 ± 2 °C, relative humidity at 55 ± 5% and a 12:12 h light-dark cycle. After acclimatization to the laboratory conditions for 1 week, the animals were randomly divided into four dietary groups of ten animals each and fed on a different experimental diet, including low fat (LF, negative control group) diet, high-fat/cholesterol (HFC, model group) diet, city diet (CD) and WR diet for 8 weeks. Body weight and food intake were routinely recorded every week throughout the experimental period. This study was approved by the Southeast University Animal Welfare Committee (Nanjing, China) and by the China Zoological Society. The protocol of the investigation was in accordance with the principles outlined in China Practice for the Care and Use of Laboratory Animals.

The experimental diets were designed, as previously described [16,19]. The powders of the composition were completely mixed, and then oil was added to the experimental diets. They were evenly made into stick strips and baked at 50–60 °C for 4–5 h. The reference LF diet was based on the AIN-76A formulation, and the HFC diet was provided with a high saturated fatty acid and cholesterol known to induce insulin resistance in rats [20,21]. For CD and WR diet, sucrose and maize starch of the HFC diet were respectively replaced with white rice and processed wheat starch or Chinese WR. The administration of the CD used in the present study, which was high in saturated fat and cholesterol, was patterned after the diet of Asian area residents in modern society [22]. The chief source of dietary carbohydrates was supplied with white rice and processed wheat starch, and many of the health-promoting components of intact whole-grain cereals were lost upon refining. Dietary carbohydrate of the WR diet was from Chinese WR, which contained all the essential parts and naturally-occurring nutrients of the entire grain seed. Chinese wild rice, which was confirmed to belong to the species *Z*. *latifolia* by the Nanjing Research Institute of Plants of the Chinese Academy of Sciences, was collected from the Luoma lakes in Eastern China. The grains were sun dried, hulled, milled into a powder, sifted through a 1mm screen and added directly to the experimental diet without pre-cooking. To ensure the food offered was completely consumed by each rat, the amount of diet offered was 10%–20% below the amount of diets consumed *ad libitum* by animals of the same age at the beginning of our studies. The amount of diet offered was increased continuously from 10.0 g at the start to 18.0 g at the end. The feeding strategy employed ensured that all rats consumed equal feed rations daily within the duration of the experiment. 

### 2.2. Tissue Collection

Blood samples were taken from the tail vein after 12 h of fasting, at the start and 4th and 6th week of the feeding period. At the 8th week, overnight fasted animals (12 h) were given mild ether anesthesia, and blood was collected from the abdominal aorta, followed by centrifugation at 3000 rpm for 10 min to separate serum. The liver and epididymal adipose tissue were immediately collected, weighed, frozen in liquid nitrogen and then stored at −80 °C until analysis. 

### 2.3. Biochemical Analysis

The concentration of fasting insulin (FINS) was determined with a radioimmunoassay kit (Chemclin Biotech Co., Ltd., Beijing, China). Serum fasting blood glucose (FBG) level, liver homogenate triglyceride (TG) and free fatty acids (FFA) concentrations were measured using commercial diagnostic kits (Jiancheng Bioengineering Institute, Nanjing, China). Homeostasis model assessment (HOMA) was employed in the present study to evaluate insulin resistance (HOMA-IR), and the index was calculated according to the reported formula [23]. Serum ADP, LCN2 and visfatin were determined by the sandwich ELISA methods using commercial rat kits, according to the manufacturer’s instructions. 

### 2.4. Reverse Transcription-Polymerase Chain Reaction (RT-PCR) Analysis

Liver and epididymal adipose tissues from all groups were collected and stored in RNAlater (Qiagen Co., Ltd., Shanghai, China) solution. Total RNA was isolated using Trizol solution (Invitrogen, USA), according to manufacturer’s instructions. Subsequently, 1 μg of total RNA of each sample was reverse transcribed into cDNA by moloney murine leukemia virus (M-MuLV) reverse transcriptase (Fermentas Co., MBI, USA). The relative gene abundances of adiponectin receptor 2 (AdipoR_2_), PPAR-α and PPAR-γ in liver tissues and leptin in epididymal tissues were quantified by the RT-PCR method in a 25 μL PCR reaction. The number of PCR cycles was 30 for each. β-actin served as an internal control. The gene expression levels were normalized to the optical density of β-actin in each sample. PCR primer sequences for each gene were synthesized commercially (Sangon Biotech, Co., Shanghai, China), and the sequences are shown in Table 1. 

**Table 1:** Sequences of primers.

Primer	Sense (5′–3′)	Antisense (5′–3′)	Size (bp)
β-actin	GAAATCGTGCGTGACATTAAG	GCTAGAAGCATTTGCGGTGGA	511
AdipoR_2_	TGCGCACACGTTTTCAGTCTCCT	TTCTATGATCCCCAAAAGTGTGC	150
PPAR-α	AGGCTATCCCAGGCTTTGC	GCGTCTGACTCGGTCTTCTTG	487
PPAR-γ	TCCGTGATGGAAGACCACTC	CCCTTGCATCCTTCACAAGC	532
Leptin	CCCATTCTGAGTTTGTCCA	GCATTCAGGGCTAAGGTC	301

### 2.5. Western Blot Analysis

The liver and epididymal adipose tissues of rats were analyzed for the protein expression of PPAR-γ and LCN2 with the Western blot technique. The sample was cooled down on ice and immediately homogenized. The protein concentration was determined according to the instructions of the Bradford kit (KeyGEN Biotech, China). Equal amounts of protein were loaded to a 10% sodium dodecyl sulfate (SDS)-polyacrylamide gel and subsequently transferred to polyvinylidene difluoride (PVDF) (Millipore Co., MA, USA) for 60 min at a 200 mA constant. Nonspecific binding sites were blocked with 5% skim milk in phosphate-buffered saline containing 0.1% Tween-20 for 60 min at room temperature. Blots were then incubated overnight at 4 °C with anti-PPAR-γ (Santa Cruz Biotechnology Co., CA, USA) or anti-LCN2 (Millipore Co., MA, USA) antibodies, according to the recommendations of the manufacturers. The antigen-antibody complexes were washed and visualized for 2 h with horseradish peroxidase-conjugated (HRP) anti-goat IgG antibody (Cell Signaling Inc., NY, USA). Antibody reactivity was detected by Enhanced Chemiluminescence (ECL) Detection Systems (KeyGEN Biotech, China). Protein loading was controlled using a monoclonal mouse antibody against β-actin antibody (Cell Signaling Inc., NY, USA). Blots were performed at least three times to confirm the reproducibility of the results. The intensity of the bands was determined by Image Tool 3.0 software and normalized with β-actin densitometric values.

### 2.6. Statistical Analysis

All data are expressed as mean ± SD. All the analyses were performed using SPSS version 13.0 statistical analysis packages (SPSS Inc., Chicago, IL, USA). The significance of difference was assessed by analysis of variance (ANOVA), followed by the Tukey *post hoc* test. Differences were considered significant at *p* < 0.05.

## 3. Results

### 3.1. Food Intake, Weight Gain, Relative Liver Weights and Liver Lipids

Throughout the experimental periods, food intake did not differ among the four diet groups (Table 2). At the fourth week, the body weights of rats fed with HFC diet (424.32 ± 15.13 g) were significantly greater than those given LF diet (409.12 ± 11.82 g) (*p* = 0.013). After eight weeks of diet feeding, the rats fed with the WR diet had significantly lower weight gain than the rats fed with the CD and HFC diet, but no significant difference was found between the CD and HFC groups. Compared to the rats fed with the LF diet, the rats fed with the HFC diet exhibited an increased relative liver weight, liver homogenate TG and FFA concentrations. The rats fed with CD showed similar relative liver weight and liver lipids, as did those fed the HFC diet. However, compared with CD, WR diet attenuated relative liver weight, liver homogenate TG and FFA levels. 

**Table 2:** Weight gain, relative liver weights and liver lipids, according to four diet groups.

	LF	HFC	CD	WR	F	*p*
Food intake (g/day)	13.63 ± 0.07	13.63 ± 0.10	13.63 ± 0.09	13.62 ± 0.10	0.11	0.954
Weight gain (g/8 weeks)	205.71 ± 19.98 ^a^	240.54 ± 15.93 ^b^	232.33 ± 20.15 ^b^	214.05 ± 20.39 ^a^	5.80	0.002
Relative liver weight (g/100 g)	2.56 ± 0.29 ^a^	4.48 ± 0.42 ^b^	4.27 ± 0.50 ^b^	2.89 ± 0.65 ^a^	39.27	<0.001
Liver lipids contents						
Triglyceride (μmol/gprot)	0.66 ± 0.28 ^a^	1.22 ± 0.19 ^b^	1.19 ± 0.13 ^b^	0.73 ± 0.19 ^a^	21.57	<0.001
Free fatty acid (μmol/gprot)	40.75 ± 4.90 ^a^	77.70 ± 10.25 ^c^	69.28 ± 9.83 ^b^	41.35 ± 7.44 ^a^	51.63	<0.001

Data are expressed as the mean ± SD (*n* = 10). ^a,b,c^ Mean values within a row, unlike superscript letters, were significantly different among four diet groups (*p* < 0.05, ANOVA followed by the Tukey *post hoc* test). LF: low-fat diet; HFC: high-fat/cholesterol diet; CD: city diet; WR: wild rice diet.

### 3.2. Fasting Blood Glucose and Insulin Levels, HOMA-IR Index

**Figure 1:**
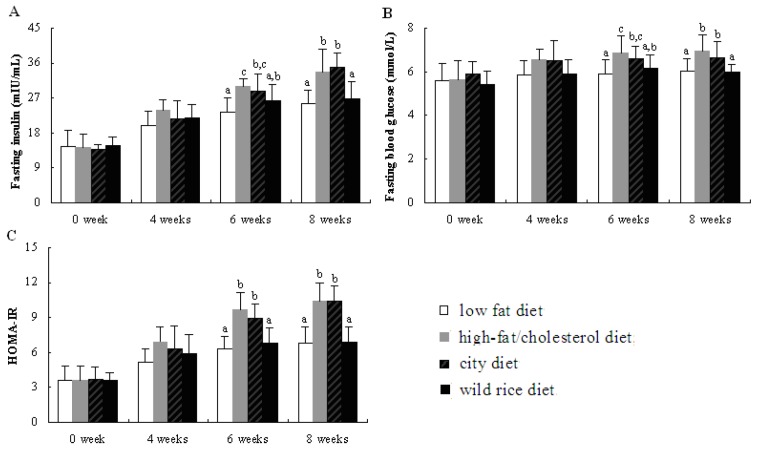
Effects of different experimental diets on glucose and insulin sensitivity in rats. Data are expressed as the mean ± SD for 10 rats. Bars without a common superscript letter indicate significant differences among groups at *p* < 0.05.

Baseline values of serum FINS (Figure 1A) and FBG (Figure 1B) were similar among the four diet groups. Compared with rats fed with CD, rats fed with the WR diet showed significant reduction in serum FINS and FBG concentrations at the end of the experiment. Both rats fed with the CD and HFC diet exhibited much lower insulin sensitivity than rats fed with the WR diet, as evidenced by the enhanced HOMA-IR index (Figure 1C). The rats fed with the WR diet showed insulin sensitivity that was comparable with the rats fed with the LF diet.

### 3.3. Serum Adiponectin, Lipocalin-2 and Visfatin Levels

There was a significant reduction in the index of serum ADP (by 46) and a significant elevation in the index of serum LCN2 (by 29) and visfatin (by 67) levels in the HFC group, as compared to those in the LF group (*p* < 0.05). The CD induced similar results in these adipocytokine levels, as it did for the HFC group. However, as compared to the CD and HFC groups, serum ADP concentration was significantly increased and serum LCN2 and visfatin concentrations were markedly decreased in the WR group (Table 3).

**Table 3:** Serum adiponectin, lipocalin-2 and visfatin concentrations, according to the four diet groups.

	LF	HFC	CD	WR	F	*p*
Adiponectin (ng/mL)	5.44 ± 0.82 ^b^	2.92 ± 0.65 ^a^	3.14 ± 0.74 ^a^	5.05 ± 0.61 ^b^	32.74	<0.001
Lipocalin-2 (pg/mL)	75.09 ± 10.98 ^a^	96.95 ± 13.38 ^b^	92.83 ± 9.98 ^b^	80.70 ± 8.49 ^a^	8.85	<0.001
Visfatin (ng/L)	32.42 ± 4.24^ a^	54.23 ± 5.14 ^c^	49.97 ± 6.19 ^c^	38.92 ± 4.25 ^b^	39.70	<0.001

Data are expressed as the mean ± SD (*n* = 10). Different superscript letters in each line indicate significant differences among groups at *p* < 0.05.^ a,b,c^ Mean values within a row, unlike superscript letters ,were significantly different among the four diet groups (*p* < 0.05, ANOVA followed by the Tukey *post hoc* test). LF: low-fat diet; HFC: high-fat/cholesterol diet; CD: city diet; WR: wild rice diet.

### 3.4. Gene Expression of AdipoR_2_, PPAR-а, PPAR-γ and Leptin in Liver or Epididymal Tissues

**Figure 2:**
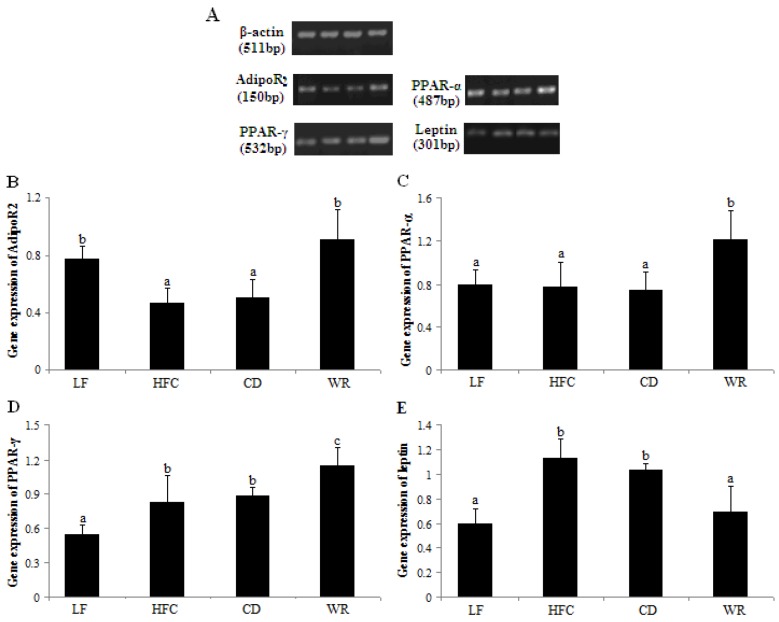
Relative mRNA abundance of AdipoR_2_ (**A**), PPAR-α (**B**) and PPAR-γ (**C**) in liver tissues and leptin (**D**) in epididymal tissues of rats fed with different experimental diets. The steady-state mRNA levels of AdipoR_2_, PPAR-α, PPAR-γ and leptin were quantified with RT-PCR and normalized against β-actin. Values are expressed as the mean ± SD for five rats. Bars without a common superscript letter indicate significant differences among groups at *p* < 0.05. LF: low-fat diet; HFC: high-fat/cholesterol diet; CD: city diet; WR: wild rice diet.

Based on band densities (Figure 2A), gene expression of AdipoR_2_ (Figure 2B) was much lower in the HFC group than in the LF group, while the gene expression of PPAR-γ (Figure 2D) and leptin (Figure 2E) were much higher in the HFC group than in the LF group. There was no significant difference in gene expression of PPAR-α (Figure 2C) between the HFC and LF groups. The CD induced similar results in the gene levels, as it did for the HFC group. Compared with the rats fed with the CD and HFC diet, the abundance of AdipoR_2_ mRNA, PPAR-α mRNA and PPAR-γ mRNA were significantly upregulated in liver tissues of rats fed with the WR diet, while the mRNA level of leptin was obviously downregulated in the epididymal tissues of rats fed with the WR diet. 

### 3.5. Protein Expression of PPAR-γ and LCN2 in Liver and Epididymal Tissues

**Figure 3:**
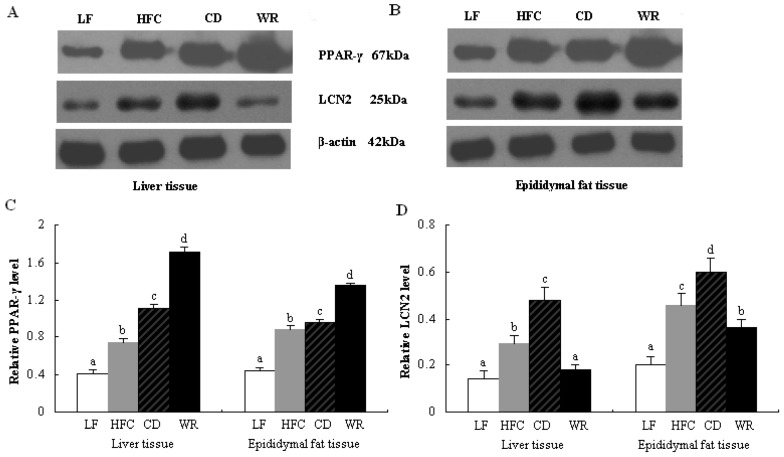
Protein expression of PPAR-γ and LCN2 in liver and epididymal adipose tissues of rats fed with different experimental diets by Western blot. The intensity of the bands was quantified by densitometric analysis and normalized with corresponding β-actin. Values are expressed as the mean ± SD for five rats. Bars without a common superscript letter indicate significant differences among groups at *p* < 0.05. LF: low-fat diet; HFC: high-fat/cholesterol diet; CD: city diet; WR: wild rice diet.

Based on band densities (Figure 3A,B), the protein expression of PPAR-γ were raised in the HFC and CD groups, compared to the LF group (Figure 3Cthe dietary carbohydrate source of CD with WR for eight weeks, protein expression of PPAR-γ was significantly higher in the WR group than in both the CD and HFC groups. In addition, both the HFC and CD diet increased protein expression of adipocytokine LCN2 relative to the LF diet (Figure 3D). After eight weeks feeding of the WR diet, protein expression of LCN2 was downregulated, compared with CD feeding. The protein expression of LCN2 in liver and epididymal tissues of the CD group was much higher than that of the HFC group.

## 4. Discussion

Extensive studies have demonstrated that whole-grain cereals rich in bioactive compounds may offer a way to control the efficiency of the metabolism of nutrients, especially those that are used as energy sources, which can decrease the development and incidence of nutrition-related chronic metabolic diseases [8,9,24]. In the present study, the protective effects of WR as a whole-grain cereal on glucose metabolism and insulin resistance were investigated in rats fed with a high-fat/cholesterol diet.

The composition of CD used in the present experimental diet was patterned after the diet of Asian area residents in modern society and was high in saturated fatty acid, cholesterol and refined carbohydrate. Most of the pericarp seed coat, aleurone and germ were removed by refining and processing in white rice and wheat starch, only leaving the starchy endosperm. Refined whole-grain wheat may lead to the loss of about 58% of fiber, 79% of vitamin E and other bioactive compounds [25]. Also, white rice and processed wheat starch had a higher rate of digestibility than whole-grain cereals, causing glycemic overload and compensatory increase in plasma insulin concentration [26]. In our study, CD was found to increase serum glucose and decrease insulin sensitivity comparable with the HFC diet, which is known to induce insulin resistance in rats [20,21]. The rats fed CD exhibited an increased weight gain and liver weight, as well as the HFC diet. In addition, both CD and HFC diet increased liver homogenate TG and FFA levels, consequently causing the occurrence of liver lipotoxicity. CD also can elevate the concentrations of serum TG and FFA in rats [19]. The CD was a hypercaloric diet, which resulted in lipid accumulation in the hepatocytes and then induced liver damage. The results taken together suggest that CD consumed by Asian area residents in modern society can lead to lipid accumulation and insulin resistance in rats, consistent with the findings of studies on Chinese urban dwellers with metabolic diseases [4].

Through replacing the dietary carbohydrate source of CD with WR, the levels of serum FBG and FINS were decreased in rats of the WR group, even when the diet consumed was high in saturated fat and cholesterol. The WR diet exerted beneficial effects on glucose tolerance and insulin sensitivity, as assessed by the HOMA-IR index. Also, the WR diet reversed the abnormal or impaired levels of liver homogenate TG and FFA levels and improved high-fat/cholesterol diet-induced lipotoxicity. The difference cannot be distributed to differential food intake and total energy intake alone, since food and energy intake throughout the feeding period were almost the same for rats that consumed CD and WR diets. In fact, the rats fed WR diet consumed slightly higher energy than the rats fed CD over the course of the experiment (12,923.2 kJ per rat fed the WR diet *vs*. 12,765.6 kJ per rat fed the CD diet). 

The effects of WR on improving insulin resistance might have accounted for the synergistic effects of its nutrient profile. For example, WR has higher dietary fiber (6.6% in the WR diet *vs*. 5.6% in the CD diet) and a lower amount of carbohydrate (43.55% in the WR diet *vs*. 44.75% in the CD diet) than common white rice and processed wheat starch. Dietary fiber can influence hormonal effects via reduction of insulin secretion, metabolic effects via increasing fat oxidation and decreasing fat storage due to greater satiety [27]. WR had three-times higher magnesium content than white rice and processed wheat starch [13,14]. Studies have shown that magnesium can improve insulin sensitivity by acting as a mild calcium antagonist and reduce the risk of type 2 diabetes [28,29]. In addition, WR lipids contain exceptional levels of hypothetically nutraceutical elements relative to that reported for regular brown rice, rice germ or wheat bran, including large amounts of phytosterols, phenolic acids, γ-oryzanols and tocotrienols [30]. WR had 10-times higher antioxidant activity than white rice, which signifies that WR prevention or attenuation of insulin sensitivity and undesirable metabolic effects is more effective than white rice and wheat starch [31]. In a word, the beneficial effects of the synergistic action of those bioactive compounds from WR were important in improving insulin resistance, since the quantity of dietary components is similar in the CD and WR diet.

Nuclear transcription factors of PPAR-α and PPAR-γ were involved in a number of biological systems, including lipogenesis, glucose and lipid metabolism, insulin sensitivity and inflammatory response [17,32,33,34]. The present study showed that gene and protein expressions of PPAR-γ were much higher in the CD and HFC groups than in the LF group. Inoue *et al*. have demonstrated that a high-fat diet can increase the expression of PPAR-γ [35]. Compared with the CD and HFC groups, WR diet significantly raised the relative expressions of PPAR-α and PPAR-γ in the liver or epididymal adipose tissues. The lipids of WR mainly contained linoleic (35%–37%) and linolenic acids (20%–31%) [30], which were natural ligands of PPAR-α and PPAR-γ [34,36]. PPAR-α activation can stimulate lipid oxidation, with a subsequent reduction of white adipose tissue depots, a decrease in ectopic lipid storage in liver and the improvement of insulin sensitivity [37], and PPAR-γ activation can enhance insulin action and increase FFA oxidation [36]. In addition, activation of nuclear transcription factors, PPAR-α and PPAR-γ, can regulate the transcription of genes involved in fatty acid synthesis and insulin sensitivity. They can also inhibit or promote the production and secretion of adipocytokines. In the present study, both the CD and HFC diet dramatically reduced serum ADP concentration and gene expression of AdipoR_2_ and raised relative expressions of LCN2 and leptin. The rats fed the WR diet exhibited an expected reverse result. WR increased the gene expression of AdipoR_2_, which can accelerate fatty acid oxidation and glucose intake [38], and WR also decreased protein expressions of LCN2, which has been implicated in the development of obesity and insulin resistance [39]. These results indicated that the mechanism of WR on high-fat/cholesterol diet-induced insulin resistance might be the activation of nuclear transcription factors, PPAR-α and PPAR-γ, and then regulation of the secretion of adipocytokines, including ADP, LCN2 and leptin. These adipocytokines work as a network to participate in diverse metabolic processes [18,40], including reducing insulin secretion and regulating insulin sensitivity. 

## 5. Conclusions

Taken together, our data demonstrate that WR has desirable properties for improving high-fat/cholesterol diet-induced insulin resistance in rats. Replacing white rice and processed wheat starch of CD with WR appears to be an effective means of preventing insulin resistance in rats fed with a high-fat/cholesterol diet. The findings suggest that WR may be used instead of white rice and processed wheat starch as a staple food in the human health diet and might serve as a potential food source for preventing nutrition-related chronic metabolic diseases.
